# Anise and grape seed oils as a feed additive to improve the performance, immune response, and antioxidant activity and reduce caecal pathogenic microbes of quail

**DOI:** 10.5194/aab-66-379-2023

**Published:** 2023-11-28

**Authors:** Alaa E. Elkomy, Amina S. El-Saadany, Effat Y. Shreif, Amal A. Bayoumi, Marwa H. Abd El-Maged, Mahmoud Alagawany, Ahmed A. Saleh, Sungbo Cho, In Ho Kim, Hossam M. Eltahan

**Affiliations:** 1 Livestock Research Department, Arid Lands Cultivation Research Institute, City of Scientific Research and Technology Applications (SRTA-City), New Borg El Arab, 21934, Egypt; 2 Faculty of Desert and Environmental Agriculture, Matrouh University, Matrouh, 51512, Egypt; 3 Animal Production Research Institute (APRI), Agriculture Research Center (ARC), Ministry of Agriculture, Dokki, 12611, Egypt; 4 Poultry Department, Faculty of Agriculture, Zagazig University, Zagazig 44519, Egypt; 5 Department of Poultry Production, Faculty of Agriculture, Kafrelsheikh University, 333516, Kafr El Sheikh, Egypt; 6 Animal Resource and Science Department, Dankook University, Cheonan, 31116, Republic of Korea

## Abstract

This trial was performed to determine the effect of anise (Ans) and grape seed (Grp) oil inclusion in diets of Japanese quail on performance, carcasses, blood parameters, antioxidant activity, and hematological blood structure. The 35 d feeding trial was conducted on two hundred 7 d old Japanese quails, allocated to four treatment groups with five replicates each. Ans and Grp were examined at different inclusion levels: 0 % (control), Ans 0.5 %, Grp 0.5 %, and Ans 0.25 % + Grp 0.25 %, in a completely randomized design. The results showed that at the end of the trial (42 d), the oil supplementation had positive effects ( P<0.001 ) on the productivity parameters, while feed intake (FI) did not differ from the control group. In addition, oil supplementation linearly improved ( P<0.05 ) the dressing percentage, carcass yield, and immune organs' relative weights, while it decreased ( p<0.01 ) the abdominal fat yield. Plasma total protein (TP), albumin (Alb), and globulin (Glob) were significantly increased in the Grp group. Despite total plasma cholesterol not being significantly affected by treatments, Ans and Grp essential oils decreased low-density lipoprotein cholesterol (LDL) and increased high-density lipoprotein cholesterol (HDL). Moreover, total antioxidant capacity (TAC) concentration was increased ( P≤0.001 ) in the treated groups, while malondialdehyde (MDA) decreased ( P≤0.001 ). Results of a caecal bacterial count revealed that Ans and its combination with Grp led to an increase ( P≤0.001 ) in *Lactobacillus* spp. count. On the other hand, Ans, Grp, and their combination led to a significant reduction ( P≤0.001 ) in *E. coli* spp. and *Salmonella* spp. compared to the control group. It is concluded that Ans and Grp seed oils could be used as valuable essential oils in quails' diets to improve the performance and immune response, enhance the antioxidant activity, and reduce caecal pathogenic microbes.

## Highlights

- Herbs and their essential oils play a crucial role in improving growth performance and gut health.
- Ans and Grp seed oils could be used as valuable essential oils in quails' diets.
- Ans and Grp seed oils improve the performance and immune response.
- Ans and Grp seed oils enhance the antioxidant activity and reduce caecal pathogenic microbes.

## 1 Introduction

For over 60 years, antibiotics have been allowed to be used in poultry feed as growth promoters without a prescription (Castanon, 2007; Abd El-Hack and Alagawany, 2022). Recently, human nutrition and diet changes have become the primary responsibility of nutrition research (Ayerza et al., 2002), and, besides the implementation of antibiotic bans in an increasing number of countries, a lot of research has been done to look for alternatives to antibiotics in the livestock industry (Abd El-Hack and Alagawany, 2022; Arain et al., 2022; Saeed et al., 2023). This ban on antibiotics in feed additives arises from the formation of resistant bacterial strains and antibiotic residues in animal products (Mehmet et al., 2005; Alagawany and Abd El-Hack, 2021). From this point of view, essential oils extracted from anise powder can improve poultry's live performance (Al-Shammari, 2011). Phytochemicals (phytogenics) are extracted from the roots, seeds, flowers, or leaves of plants, and their most familiar activities are linked to the density of phenolic terpenes or terpenoids, flavonoids, and anthocyanins (Rafeeq et al., 2023). Many of these compounds show antibacterial activity against various pathogens (Dorman and Deans, 2000). Herbs, including spices, medicinal herbs, and their essential oils, play a crucial role in improving growth performance and gut health in poultry according to their active ingredients with safe, low-toxicity, and residue-free properties (Aziza and Cherian, 2010; Righi et al., 2021) and boost digestive enzyme production and enhance digestive products' utilization (Jamroz and Kamel, 2002; Ramakrishna et al., 2003; Hernandez et al., 2004). Moreover, several natural plants' antioxidant compounds have been specified as free radicals or active oxygen scavengers (Dündar, 2001) and are used to reduce the oxidation of polyunsaturated fats in foods (Şahin et al., 2010; Mahfuz et al., 2021).

Very little is known about the role of aniseed on oxidant–antioxidant balance. Anise (*Pimpinella anisum* L.) is an Apiaceae family annual essential herb, and its seeds are valuable (Al-Beitawi et al., 2009). Previous studies by Gülçın et al. (2003) reported that grape seed essential oil consists of methyl chavicol, eugenol trans-Anethole, coumarins, estragole, anisaldehyde, scopoletin, estrols, umbelliferone, polyenes, terpene hydrocarbons, and polyacetylenes. These have antioxidant, anti-microbial, analgesic, and insecticidal pharmacological properties (G. W. Wang et al., 2011). Essential oil (2 %–6 %), fat (30 %), proteins (20 %), gum (6 %–7 %), minerals (6 %–7 %), pentosan (5 %–7 %), sugars (4 %), and furfural (3 %) are the main components of aniseed, in addition to phenolic acids and flavonol and flavone glycosides. Moreover, anethole makes up 80 %–95 % of the essential oil (Franz et al., 2005). According to Al-Shammari (2011) and Ertas et al. (2005), aniseed positively affect the digestibility of nutrients; enhance the digestion of protein, cellulose, and fat; and improve the ileal digestibility of nutrients.

Studies on grape seeds are limited, despite their richness in polyphenolic substances, especially monomeric and oligomeric flavanols (Brenes et al., 2008). Guangtian et al. (2020) mentioned that grape seed is a source of vitamin E and flavonoids (especially proanthocyanidins) that have a potential effect as an antibiotic substitute in broilers. Grape seed proanthocyanidin extract (GSPE) enhanced the growth performance of broilers by strengthening antioxidant capacity, improving immune responses, and developing short-chain fatty acids of the cecum. In the same context, thanks to its capability to absorb oxygen radicals, GSPE is widely recognized as a potent antioxidant with anti-inflammatory and anti-cancer properties (Ouédraogo et al., 2011; Sayin et al., 2014). GSPE provides more protective benefits against free radicals, free-radical-induced lipid peroxidation, and DNA damage than vitamins C, E, and A. (Bagchi et al., 2000; Xu et al., 2015). Furthermore, proanthocyanidins are possible antibacterial agents that can inhibit the colonization of pathogenic intestinal bacteria (Papadopoulou et al., 2005; Viveros et al., 2011). Including polyphenol grape by-products in animal feed decreased lipid peroxidation in meat and efficiently altered intestinal flora (Brenes et al., 2016). Therefore, we hypothesized that using anise (Ans) and grape seed (Grp) in quail chicks' nutrition as a natural growth-promoting substance will enhance quail performance, blood constituents, redox status, and caecal microbial count.

## 2 Material and methods

### 2.1 Animals and house management

This study was carried out at the Poultry Farm, Faculty of Desert and Environmental Agriculture, Matrouh University. This trial was performed on two hundred 7 d old mixed-sex quails (*Coturnix coturnix japonica*) with an initial body weight of 35 ± 0.5 g. Using a completely randomized design, the quails were individually weighed and randomly allotted to four treatment groups of 50 quails each (five replicates per group). The experiment lasted for 35 d. Birds were reared in a caged wire floor battery in a controlled environmental house with 23 h of light per day with a luminous intensity of 10 lux during the 5-week experimental period. The environmental temperature was about 32 ${}^{\circ }$ C during the first week; about 2 ${}^{\circ }$ C was gradually reduced weekly until about 24 ${}^{\circ }$ C in the fourth week up to the end of the experiment (at 42 d of age).

### 2.2 Management and diets

Different levels of anise (Ans) and grape (Grp) seeds oils, were studied at 0 % (control), Groups 2 and 3 were fed basal diets supplemented with 0.5 % aniseed oil (Ans) and 0.5 % grape seed oil (Grp), respectively, while in group 4, the diet was supplemented with 0.25 % Ans + 0.25 % Grp. Experimental diets were formulated as isocaloric–isonitrogenous, which provided chicks with 24 % protein and 3000 kcal kg ${}^{-1}$ feed in the shape of pellets. Feed and water were provided ad libitum throughout the experimental period (from 7–42 d of age). According to the NRC (1994), diets were formulated to meet their needs. The diet ingredients included in the current experiment are listed in Table 1.

**Table 1:** Feed ingredients and calculated analysis of the basal diet for growing quails. DDGS: distiller's dried grains with soluble.

Ingredients	g/100 g
Yellow corn (8.2 %)	53.450
Soybean meal (48 %)	27.600
Corn gluten meal (62 %)	5.150
DDGS (31 %)	9.525
Soybean oil	0.825
Limestone	0.800
Dicalcium phosphate	1.750
Lysine	0.200
Methionine	0.100
Salt	0.300
Premix ${}^{\text{a}}$	0.300
Total	100.000
Chemical analysis	
Protein (%)	24.000
Metabolizable energy (kcal kg ${}^{-1}$ )	2987.0
Crude fiber (%)	3.070
Crude fat (%)	3.570
Lysine (%)	1.300
Calcium (%)	0.800

${}^{\text{a}}$ Each kilogram of premix mixture contained: vitamin A 10 000 000 IU; vitamin D3 3 000 000 IU; vitamin E 2500 Mg; vitamin K3 4000 Mg; vitamin B1 5000 Mg; vitamin B2 500 Mg; vitamin B6 2500 Mg; vitamin B12 5 Mg; vitamin C 10 000 Mg; nicotinamide 20 000 Mg; pantothenic acid 5000 Mg; biotin 5 Mg; folic acid 250 Mg; D. L methionine 10 000 Mg; L. lysine 5000 Mg; Fe 70 Mg; CuSO ${}_{4}$ 120 Mg; ZnSO ${}_{4}$ 60 Mg; MnSO ${}_{4}$ 160 Mg; CoSO ${}_{4}$ 16.2 Mg; MgSO ${}_{4}$ 70 Mg; calcium iodate 50 Mg; NaCl 7000 Mg and KCl 15 000 Mg.

### 2.3 Data collected

#### 2.3.1 Growth performance

Each treatment group recorded each bird's live body weight (LBW) and weight gain (BWG) weekly. Feed intake (FI) and feed conversion ratio (FCR) (as feed intake/body weight gain) were recorded at 0, 3, and 6 weeks of age.

#### 2.3.2 Carcass traits

Ten birds per treatment (equal distribution between males and females in each group) were chosen and slaughtered at 42 d of age to determine carcass weight and inner body organ weight (gizzard, liver, spleen, thymus, bursa, and abdominal fat) as a relative to live body weight.

#### 2.3.3 Blood sampling and blood biochemical constituents

At the end of the experiment period, blood samples of the groups were collected from birds during scarifying. Fresh blood samples were used to determine blood parameters, including the red blood cell counts (RBCs) and white blood cell counts (WBCs) and differentiation (lymphocytes % and heterophils %), hemoglobin (Hb, g dL ${}^{-1}$ ), and hematocrit (PCV). Heparin was utilized as an anticoagulant; however, serum was extracted from specific samples without heparin. Blood samples were centrifuged at 3500 rpm for 20 min to extract plasma or serum, which was then frozen at -20 ${}^{\circ }$ C for further biochemical analysis. Plasma total protein (TP), albumin (Alb), and globulin (GLOB) concentration as (g dL ${}^{-1}$ ) were measured. Plasma total cholesterol (TCHO), high-density lipoprotein cholesterol (HDL), low-density lipoprotein cholesterol (LDL), and triglyceride (TG) concentrations as (mg dL ${}^{-1}$ ) were determined spectrophotometrically using commercial kits from Biodiagnostic Company (Giza, Egypt). Total antioxidant capacity (TAC) as (mmol L ${}^{-1}$ ) and malondialdehyde (MDA) as ( µ mol mL ${}^{-1}$ ) as well as glutathione peroxidase (GPx) concentrations and superoxide dismutase (SOD) activities were determined in plasma using commercial kits and a spectrophotometer (Shimadzu, Japan).

#### 2.3.4 Cecal microbiota

The carcasses were cut open, and the whole gastrointestinal tract was aseptically removed. Before detaching the ceca from the tract intestine, the tract was separated into parts that were ligated with light thread. Afterward, they were preserved in sterile bags containing 50 mL of ice-cold cryoprotective broth, which was utilized to protect gut flora (Ballongue et al., 1997) and stored at -80 ${}^{\circ }$ . The digesta from the cecum was frozen and transferred to a new sterile bag for bacterial counting. Cecal digesta was immediately diluted 10-fold (i.e., 10 % w/v ) with sterile ice-cold anoxic phosphate-buffered saline (PBS) (0.1 M; pH 7.0) and homogenized for 3 min. Each homogenate of the cecum was serially diluted from 101 to 107. Subsequently, dilutions were plated in duplicate on selective agar media for target bacteria groups, and the enumeration results were expressed as colony-forming units (CFUs) log ${}_{10}$ per milliliter. Plate count agar, MRS agar for LAB, MacConkey agar media for Lactobacillus spp., and *E. coli* and Shigella agar plates were used for *Salmonella* counts (Tuohy et al., 2002). The plates were then incubated at 37 ${}^{\circ }$ C for 24 to 72 h. The colony-forming units per gram of the cecal digest were calculated using a base-10 logarithm.

#### 2.3.5 Statistical analysis

The trial was performed using a completely randomized design with four experimental groups of five replicates each. The normality of the data was checked by the Kolmogorov–Smirnov test before the statistical analysis. Data were analyzed by One-way ANOVA using IBM SPSS statistical package (version 22, SPSS Inc., Chicago, IL, USA) to evaluate the effect of treatment, along with a Tukey's test ( P<0.05 ). The replicate pen was used as an experimental unit for the performance data, and the bird for the other variables. Significance was set at ( P<0.05 ), and values are presented as means ± standard error of the mean (SEM).

**Table 2:** Effect of Ans and Grp oil supplementation on the growth performance of growing quails.

Trait	Control	Ans oil	Grp oil	Ans + Grp oil	P value
		0.5 %	0.5 %	0.25 + 0.25 %	
Initial body weight (7 d), g	35.67 ± 0.71	35.36 ± 0.73	35.61 ± 0.74	35.97 ± 0.39	0.933 ${}^{\text{NS}}$
Final body weight (42 d), g	193.82 ${}^{\text{c}}$ ± 4.1	215.5 ${}^{\text{ab}}$ ± 6.4	205.5 ${}^{\text{bc}}$ ± 4.8	226.88 ${}^{\text{a}}$ ± 5.3	0.000 ${}^{**}$
Body weight gain, g per bird per 7–42 d	158.15 ${}^{\text{c}}$ ± 3.9	177.15 ${}^{\text{b}}$ ± 5.3	169.9 ${}^{\text{bc}}$ ± 4.3	190.9 ${}^{\text{a}}$ ± 5.2	0.000 ${}^{**}$
Feed intake, g per bird per 7–42 d	460.87 ± 3.33	455.41 ± 1.67	454.71 ± 1.96	461.09 ± 1.43	0.081 ${}^{\text{NS}}$
Feed conversion ratio, g feed per g weight gain	2.92 ${}^{\text{a}}$ ± 0.02	2.57 ${}^{\text{b}}$ ± 0.02	2.67 ${}^{\text{b}}$ ± 0.05	2.42 ${}^{\text{c}}$ ± 0.08	0.000 ${}^{**}$

${}^{\text{NS}}$ Non-significant. ${}^{*}\;P\leq 0.05$ . ${}^{**}\;P\leq 0.01$ . ${}^{\text{a,b,c}}$ Mean values with different superscripts within a row differ significantly.

## 3 Results and discussion

### 3.1 Growth performance

At the end of the experiment period, Table 2 indicated that the chick's LBW was higher in all treated groups versus the control group. This improvement was highly significant ( P≤0.01 ) with Ans + Grp oil mixture (226 g) followed by Ans oil (215 g), while the Grp oil group (205 g) was comparable to the control group (193 g). Results of BWG during age 7–42 d had approximately the same trend as LBW, whereas Ans and Ans + Grp oils resulted in a significant increase ( P≤0.01 ) of BWG in contrast to the control group with a preference for the Ans + Grp oil mixture. Our findings agreed with those of prior research that mentioned adding Ans powder in broiler chicks' diet up to 1 % (Al-Kassie, 2008) or up to 1.5 g kg ${}^{-1}$ (Muhammad et al., 2014) led to a significantly improve the performance of birds based on LBW and BWG, similar results were found by Mohammed (2019). A total of 400 mg Ans oil kg ${}^{-1}$ feed as a natural growth-promoting substance significantly increased daily LBW. This increase may be due to active ingredients such as anethole, eugenol, methyl chavicol, anisaldehyde, and estragole in anise (Mehmet et al., 2005). Mainly, anethole and eugenol have digestive-stimulating effects and showed an increasing influence on live weight gain and feed conversion (Çabuk et al., 2003). Several studies illustrated that anise essential oil has anti-microbial (Singh et al., 2002; Tabanca et al., 2003), antifungal (Soliman and Badea, 2002), antiparasitic (Çabuk et al., 2003), and antipyretic effects (Afifi et al., 1994). Moreover, it enhanced protein, cellulose, and lipid digestion (Jamroz and Kamel, 2002), improved whole-tract and ileal nutrient absorption digestibility (Hernandez et al., 2004), and raised the impacts of pancreatic lipase and amylase (Ramakrish et al., 2003). Moreover, there is evidence that the active compounds of cumin (2-methoxy-3-s-butyl pyrazine and 2-methoxy-3-methylpyrazine) enhanced bile acid concentration and motivated the pancreas and stimulated the secretion of digestive enzyme activities, including amylases proteases and lipases (Srinivasan, 2005; Saleh et al., 2019).

On the other hand, the results of Grp oils on the Japanese quail growth performance agreed with the results of Wang et al. (2008), who concluded that adding 10 to 20 mg kg ${}^{-1}$ of grape seed extract to broiler diets improved growth performance. Farahat et al. (2017) reported that adding 125 and 250 mg kg ${}^{-1}$ grape seed extract (GSE) to broiler diets resulted in insignificant improvement in performance compared to the control. They illustrated that this non-significant improvement might be due to the GSE's low dosages. Abu Hafsa and Ibrahim (2018) confirmed that grape seed extract could be used as a herbal supplement in broiler diets to enhance LBW and BWG. The results of Guangtian et al. (2020) suggest that grape seed phenolic compounds, mainly Proanthocyanidins were likely to improve the health status and increase the average daily weight gain (DWG) of birds.

Previous studies on GSPE revealed that the addition of GSPE significantly reduced birds' average daily feed intake (DFI; Guangtian et al., 2020). Viveros et al. (2011) discovered that treatment with polyphenol-rich grape pomace extract boosted broilers' FCR ratio and nutrient digestibility by increasing the intestinal absorption surface. Also, GSPE could treat the lesion caused by aflatoxin B1 and significantly improve the average DFI, DWG, and FCR compared to the aflatoxin B1 group (Long et al., 2016; Rajput et al., 2019). Generally, Studies demonstrated that essential oils boosted protein, cellulose, and fat digestion (Jamroz and Kamel, 2002), ileal digestibility of the nutrients (Hernandez et al., 2004), and the actions of pancreatic lipase and amylase (Ramakrishna et al., 2003).

Throughout the experimental period, adding Ans, Grp, or their mixture to quail chicks' diet did not affect FI, whereas all treated groups consumed almost the same amount of feed. In addition, Jamroz and Kamel (2002) and Ramakrishna et al. (2003) showed non-significant variations in rat feed consumption between aniseed-supplemented and unsupplemented groups. In contrast to the findings of Guo et al. (2004), who saw an increase in feed intake in the aniseed-supplemented groups, our results indicate no such increase. Generally, the present results showed an advantage of using essential oils on FCR, where FCR improved significantly ( P≤0.01 ) in all treatment groups relative to the control group, with a preference for the Ans + Grp combination group. The results of improved FCR by anise treatments correspond with the results of Mehmet et al. (2005), Al-Kassie (2008), Muhammad et al. (2014), and Mohammed (2019). The improvement in growth performance traits may be attributable to anethole, an active component in aniseed that stimulates the digestive system (Tucker, 2002; Giannenas et al., 2003) and positively affects the digestibility of nutrients. Likewise, Mehmet et al. (2005) and Al-Shammari et al. (2017) mentioned that aniseed's digestive stimulating and antibacterial activities could account for its growth-promoting properties. In addition, Caiyun et al. (2019) found that chickens fed diets enriched with star anise oil at doses of 200, 400, and 600 mg kg ${}^{-1}$ had higher metabolic efficiency of crude protein, organic matter, lysine, methionine, and arginine than those of the control group.

**Table 3:** Effect of Ans and Grp oil supplementation on relative carcass and organ weight of growing quails.

Trait	Control	Ans oil	Grp oil	Ans + Grp oil	P value
		0.5 %	0.5 %	0.25 + 0.25 %	
Carcass, %	71.02 ${}^{\text{c}}$ ± 0.10	78.12 ${}^{\text{ab}}$ ± 0.12	75.75 ${}^{\text{bc}}$ ± 0.54	81.51 ${}^{\text{a}}$ ± 0.23	0.000 ${}^{**}$
Gizzard, %	2.55 ± 0.015	2.60 ± 0.007	2.57 ± 0.019	2.58 ± 0.01	0.087 ${}^{\text{NS}}$
Liver, %	2.46 ± 0.015	2.45 ± 0.018	2.44 ± 0.035	2.46 ± 0.050	0.959 ${}^{\text{NS}}$
Spleen, %	0.065 ± 0.002	0.066 ± 0.005	0.067 ± 0.004	0.066 ± 0.007	0.998 ${}^{\text{NS}}$
Thymus, %	0.153 ${}^{\text{d}}$ ± 0.002	0.183 ${}^{\text{b}}$ ± 0.002	0.167 ${}^{\text{c}}$ ± 0.004	0.207 ${}^{\text{a}}$ ± 0.006	0.000 ${}^{**}$
Bursa, %	0.140 ${}^{\text{b}}$ ± 0.004	0.167 ${}^{\text{a}}$ ± 0.002	0.163 ${}^{\text{a}}$ ± 0.006	0.167 ${}^{\text{a}}$ ± 0.002	0.000 ${}^{**}$
Abdominal fat, %	1.13 ${}^{\text{a}}$ ± 0.092	0.70 ${}^{\text{b}}$ ± 0.063	0.73 ${}^{\text{b}}$ ± 0.021	0.67 ${}^{\text{b}}$ ± 0.042	0.000 ${}^{**}$

${}^{\text{NS}}$ Non-significant. ${}^{*}\;P\leq 0.05$ . ${}^{**}\;P\leq 0.01$ . ${}^{\text{a,b,c}}$ Mean values with different superscripts within a row differ significantly.

### 3.2 Carcass characteristics

Data on slaughter traits and their statistical analysis are displayed in Table 3. Relative carcass weights were significantly elevated ( P≤0.01 ) in all groups treated with essential oils versus the control group. The Ans + Grp oil mixture group had the highest carcass relative weight. In addition, the relative weights of the gizzard, liver, and spleen in 42 d old quail chicks indicated that none of the treatment groups differed significantly from the control group. On the other hand, the relative weights of the thymus and bursa glands were significantly improved ( P≤0.01 ) in all treated groups, and the Ans + Grp oil mixtures had the highest values. The inclusion of Ans, Grp or their mixture in quail chicks' rations resulted in decreasing body lipid deposition, whereas relative abdominal fat deposit tissues weight was significantly decreased ( P≤0.01 ) in all treated groups compared to the control group, and this decrease was 38 %, 35 %, and 40 % less than in the control group for Ans, Grp, and Ans + Grp oil mixture groups, respectively.

Increasing LBW observed in groups treated with essential oils compared to the control group was reflected in an increase in their relative carcass weight. This result may be due to enhancing the nutrient digestibility and absorption through the digestive tract, which was reflected in increased protein deposit in tissue. The previous studies revealed that adding aniseed powder or aniseed oil to the broiler's diet positively affected carcass weight, and the dressing percentage was significantly higher than in the control group (Muhammad et al., 2014; Mohammed, 2019). Also, essential oils derived from different essential plants have improved feed conversion and carcass yield (Tucker, 2002). This improvement might be due to active ingredients such as anethole, eugenol, methyl chavicol, anisaldehyde, and estragole in anise. Especially anethole and eugenol have stimulating digestive effects (Çabuk et al., 2003); besides, anethole affected pathogen microorganisms in the digestive system. In the same context, Turcu et al. (2021) found that the inclusion of Grp oil in broiler rations at 1.5 % and 3 % resulted in a higher carcass percentage. Muhammad et al. (2014) mentioned that treating broilers with aniseed resulted in a significant increase in the absolute weight of various organs (liver, kidney, thymus, and spleen) in the fourth week of age compared to the control. Although Zou et al. (2006) determined that the inclusion of plant extracts had a significantly favorable influence on spleen and thymus weights, Simsek et al. (2007) and Hernandez et al. (2004) illustrated that providing aniseed extract to broiler chicks did not affect the weight of the spleen.

**Table 4:** Effect of Ans and Grp oil supplementation on blood hematological parameters of growing quails.

Trait	Control	Ans oil	Grp oil	Ans + Grp oil	P value
		0.5 %	0.5 %	0.25 + 0.25 %	
RBC, 10 ${}^{6}$ mm ${}^{-3}$	3.12 ± 0.07	3.13 ± 0.09	3.39 ± 0.10	3.10 ± 0.07	0.062 ${}^{\text{NS}}$
Hb, g dL ${}^{-1}$	10.37 ${}^{\text{b}}$ ± 0.09	10.57 ${}^{\text{b}}$ ± 0.08	12.43 ${}^{\text{a}}$ ± 0.55	11.37 ${}^{\text{ab}}$ ± 0.34	0.001 ${}^{**}$
PCV, %	32.67 ${}^{\text{b}}$ ± 0.33	33.33 ${}^{\text{b}}$ ± 0.53	37.10 ${}^{\text{a}}$ ± 0.40	35.67 ${}^{\text{a}}$ ± 0.26	0.000 ${}^{**}$
WBC, 10 ${}^{3}$ mm ${}^{-3}$	17.23 ${}^{\text{c}}$ ± 0.12	18.83 ${}^{\text{b}}$ ± 0.15	18.40 ${}^{\text{b}}$ ± 0.04	19.70 ${}^{\text{a}}$ ± 0.13	0.000 ${}^{**}$
Lymphocytes, %	47.23 ${}^{\text{c}}$ ± 0.63	50.49 ${}^{\text{b}}$ ± 0.25	51.08 ${}^{\text{b}}$ ± 0.70	54.74 ${}^{\text{a}}$ ± 0.10	0.000 ${}^{**}$
Heterophils, %	27.79 ${}^{\text{a}}$ ± 0.05	24.65 ${}^{\text{b}}$ ± 0.30	23.57 ${}^{\text{b}}$ ± 0.48	24.99 ${}^{\text{b}}$ ± 0.57	0.000 ${}^{**}$
Het / Lym ratio	0.59 ${}^{\text{a}}$ ± 0.01	0.49 ${}^{\text{b}}$ ± 0.01	0.46 ${}^{\text{b}}$ ± 0.02	0.45 ${}^{\text{b}}$ ± 0.06	0.000 ${}^{**}$

${}^{\text{NS}}$ Non-significant. ${}^{*}\;P\leq 0.05$ . ${}^{**}\;P\leq 0.01$ . ${}^{\text{a,b,c}}$ Mean values with different superscripts within a row differ significantly.

### 3.3 Blood hematological and biochemical parameters

Results presented in Table 4 showed that the inclusion of Ans, Grp, or their mixture in quail chicks' diets did not affect the RBC, and the RBC count for the treatment groups was not significantly different from the control group. Despite the RBC count not being affected by adding essential oils to chicks' rations, the Hb and PCV were increased in the groups treated with essential oils compared to in the control group; this rise was significant ( P≤0.01 ) with Grp and Ans + Grp mixture only. Chicks fed a ration supplemented with Ans, Grp, or Ans + Grp oil mixtures exhibited a substantial increase ( P≤0.01 ) in WBC and the percentage lymphocytes (Lym %) compared to the control group. Within essential oil treatments, the highest WBC and lymphocyte (Lym) values were noted for the Ans + Grp mixture treatment, while the Ans treatment was identical to the Grp treatment. In contrast, the percentage of heterophils (Het) was significantly decreased in groups treated with Ans, Grp, or Ans + Grp oil mixtures compared to the control group. Increasing Lym and decreasing Het percentages of quail chicks fed the diet with essential oil supplementation reflected a significantly reducing Het / Lym ratio relative to the control group. Our data showed that the blood antioxidant level (TAC and the activity of glutathione peroxidase (GSH-px) and SOD) was boosted (Fig. 2). The level of malondialdehyde (MAD) was decreased in groups that received an Ans- or Grp-oil-supplemented diet. This effect was due to Ans and Grp oils containing many antioxidant compounds characterized as active oxygen scavengers or free radicals and maintaining the intracellular redox balance. The free-radical scavenging effect of Ans and Grp was reflected in improving the quail chicks' health and alleviating stress, which resulted in the reduction in the Het / Lym ratio in treated groups compared to the control group. According to Gross and Siegel (1983), the Het / Lym ratio appears to be a more valuable tool than corticosterone serum levels for explaining the many stress variables to which birds are exposed. Al-Shammari et al. (2017) discovered that aniseed powder at 0, 500, 750, and 1000 mg L ${}^{-1}$ in broiler drinking water significantly increased RBC, WBC, and Hb levels while decreasing the Het / Lym ratio.

**Table 5:** Effect of Ans and Grp oil supplementation on blood biochemical parameters of growing quails.

Trait	Control	Ans oil	Grp oil	Ans + Grp oil	P value
		0.5 %	0.5 %	0.25 + 0.25 %	
Total protein, g dL ${}^{-1}$	3.04 ${}^{\text{b}}$ ± 0.116	3.13 ${}^{\text{b}}$ ± 0.077	3.15 ${}^{\text{b}}$ ± 0.077	3.93 ${}^{\text{a}}$ ± 0.029	0.000 ${}^{**}$
Albumin, g dL ${}^{-1}$	1.70 ${}^{\text{b}}$ ± 0.039	1.71 ${}^{\text{b}}$ ± 0.032	1.73 ${}^{\text{b}}$ ± 0.050	2.19 ${}^{\text{a}}$ ± 0.027	0.000 ${}^{**}$
Globulin, g dL ${}^{-1}$	1.34 ${}^{\text{b}}$ ± 0.085	1.43 ${}^{\text{b}}$ ± 0.040	1.43 ${}^{\text{b}}$ ± 0.114	1.74 ${}^{\text{a}}$ ± 0.007	0.005 ${}^{**}$
Cholesterol, mg dL ${}^{-1}$	159.67 ± 0.56	154.33 ± 3.51	153.67 ± 1.48	152.67 ± 1.56	0.115
HDL, mg dL ${}^{-1}$	41.67 ${}^{\text{c}}$ ± 0.21	46.67 ${}^{\text{b}}$ ± 0.56	46.67 ${}^{\text{b}}$ ± 0.42	49.00 ${}^{\text{a}}$ ± 0.37	0.000 ${}^{**}$
LDL, mg dL ${}^{-1}$	90.20 ${}^{\text{a}}$ ± 0.25	80.87 ${}^{\text{b}}$ ± 3.98	80.20 ${}^{\text{b}}$ ± 1.32	78.00 ${}^{\text{b}}$ ± 1.51	0.005 ${}^{**}$
Triglycerides, mg dL ${}^{-1}$	139.00 ${}^{\text{a}}$ ± 0.97	134.00 ${}^{\text{b}}$ ± 0.73	134.00 ${}^{\text{b}}$ ± 1.27	128.33 ${}^{\text{c}}$ ± 0.21	0.000 ${}^{**}$

${}^{*}\;P\leq 0.05$ . ${}^{**}\;P\leq 0.01$ . ${}^{\text{a,b,c}}$ Mean values with different superscripts within a row differ significantly.

Table 5 displays the impact of the inclusion of Ans, Grp, or their mixture on blood traits in quail chicks. Ans + Grp oil mixtures in quail chicks' diets resulted in significant increases in TP ( P≤0.01 ), Alb ( P≤0.01 ), and globulin (Glob; P≤0.05 ) in contrast to the control diet or Ans and Grp oils groups. The TCHO of the three treated groups decreased compared to the control group. The LDL level was decreased in the treatment groups compared with those in the control group ( P≤0.05 ). Despite the total cholesterol level being non-significantly reduced, HDL concentration rose significantly ( P≤0.01 ) in treated groups compared to untreated groups; meanwhile, the Ans + Grp oil mixture significantly boosted plasma HDL more than the Ans or Grp alone. Feeding quail chicks an essential oil treatment significantly reduced ( P≤0.01 ) plasma TG (mg dL ${}^{-1}$ ) concentration compared to the control, and the maximum effect was obtained in the Ans + Grp oil mixture treatment. Reducing birds' blood TCHO, LDL, and TG and increasing HDL levels are essential for the safety of poultry product consumers. Our results are in harmony with Salman's finding (2019), which showed that Grp oil, at levels 1 % and 2 %, conducted a highly significant increase in broilers' TP and Glob and HDL levels compared with the control treatment. By contrast, TCHO, LDL, cholesterol (Chol), and TG levels significantly decreased ( P≤0.01 ).

**Figure 1:**
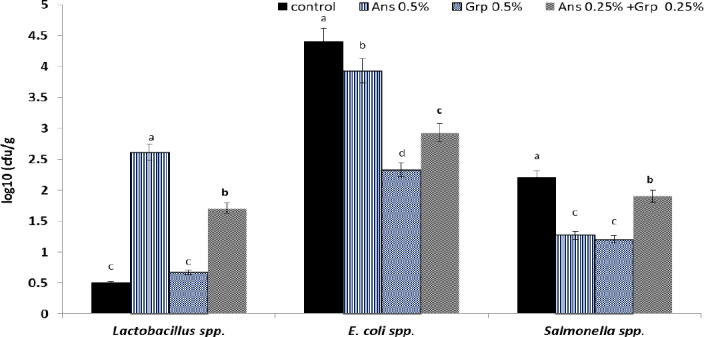
Caecum microbial count (log ${}_{10}$ ; CFU g ${}^{-1}$ ) of growing quails treated with levels of Ans oil (0.50 %), Grp oil (0.50 %), or their mixture (0.25 Ans + 0.25 % Grp). The values are represented by vertical bars. ${}^{\text{a,b,c}}$ Mean values with different letters were different ( P≤0.05 ).

Additionally, according to results found by Brenes et al. (2010), diets containing 250 and 500 mg kg ${}^{-1}$ GSE significantly enhanced TP, Alb, and Glob in broiler chicken. By contrast, Kaya et al. (2014) showed that serum TP and Alb levels of hens fed 2025 mg GSE kg ${}^{-1}$ feed were lower than those of hens on a control diet. Grape seed oil possesses numerous pharmacological activities, including characteristics against the oxidation of low-density lipoproteins and the lowering of serum Chol (Cao and Ito, 2003). And this reduction may be connected with the polyphenolic chemicals in grape seed and the structure of the insoluble fiber, which enhances lipid excretion, which may, in turn, reduce the Chol level. In rats fed grape seed proanthocyanidins, El-Alfy et al. (2005) also noticed a reduction in hyperglycemia and an increase in serum insulin levels. Fakhraddin and Habib (2014) revealed that grape pomace could be included in the diets of broilers to decrease Chol and TG in plasma.

Our results were consistent with the findings of Al-Shammari et al. (2017), who reported that including aniseed powder in drinking water at concentrations of 0, 500, 750, and 1000 mg L ${}^{-1}$ significantly increased blood TP, Alb, and Glob concentrations of broilers in contrast to the control group while decreasing the levels of TCHO, TG, and total lipids. Christaki et al. (2012) also discovered that serum TCHO and TG levels were significantly reduced in Japanese quails treated with powdered aniseed at a 10 and 20 g kg ${}^{-1}$ diet.

The significant reductions in triglycerides and cholesterol levels by feeding diets supplemented with Ans and Grp seed oil may be because of their high content of unsaturated fatty acids, particularly linoleic acid (Abdel-Aal and Attia, 1993). Linoleic acid may stimulate cholesterol excretion into the intestine (Tollba and Hassan, 2003). Nonetheless, the increase in total protein and albumin may be because of an immuno-stimulating effect of Ans and Grp seed oil (Aqel, 1993). Ans and Grp seed oil contain reasonable amounts of macro- and micro-mineral elements that are involved in the growth process. They are essential for optimal performance and could enhance the immune system and increase total protein and albumin concentrations (William, 1997).

**Figure 2:**
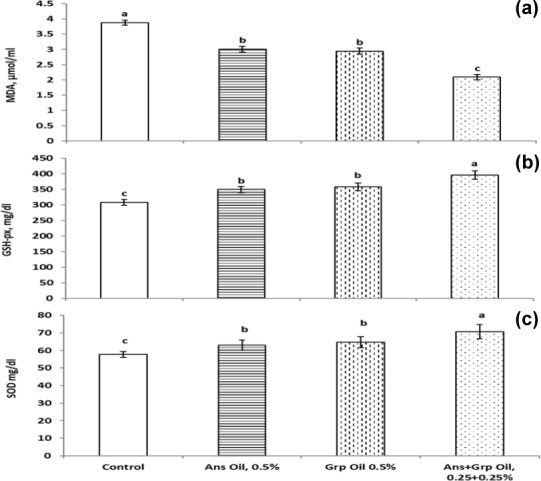
Antioxidant activity of growing quails treated with levels of Ans oil (0.50 %), Grp oil (0.50 %), or their mixture (0.25 Ans + 0.25 % Grp). The values are represented by vertical bars. ${}^{\text{a,b,c}}$ Mean values with different letters were different ( P≤0.05 ).

## 4 Cecal microbial count

Results presented graphically in Fig. 1 illustrated the cecal microbial count (log ${}^{10}$ (CFU g ${}^{-1}$ )). The Ans oil inclusion in the quail diet enhanced the environmental conditions for the presence of *Lactobacillus* bacteria in quail caecum, whereas the *Lactobacillus* count increased significantly ( P≤0.01 ) compared to the control group. However, the *Lactobacillus* count in the Grp oil group was similar to the control group; The Ans + Grp mixture increased the *Lactobacillus* count significantly in comparison to the control; still, this increase was less than in Ans oil alone. Adding Ans, Grp, or their mixture led to a significant reduction ( P≤0.01 ) in caecum *E. coli* count; however, the Grp essential oil had the highest effect, followed by the Grp + Ans mixture and then Ans oil. The results for the *Salmonella* spp. count in quail caecum revealed a significant reduction ( P≤0.01 ) in the treatment groups.

Interestingly, the results showed that Grp oil significantly decreases pathogenic microbes (such as *E. coli* and *Salmonella* spp.) but does not enhance the beneficial bacteria growth. By contrast, Ans oil significantly improved *Lactobacillus* spp. growth, but it has a weak effect as an anti-pathogen. Anhê et al. (2015) reported that polyphenols derived from various plants helped alter the composition of the gut microbiota, which raised the number of *Lactobacillus* spp. Anise oil and its primary constituent, anethole, have demonstrated antibacterial efficacy against aerobic and anaerobic bacterial growth (Gülçın et al., 2003; J. P. Wang et al., 2011), and they have an effect on pathogen microorganisms in the digestive tract (Çabuk et al., 2003).

Furthermore, grape by-products as antibacterial agents are the subject of much dispute, and feed additives altered the gastrointestinal microbiota of broilers (Barcelo et al., 2000; Xia et al., 2010). According to research, the flavonoids in grape by-products have been shown to inhibit pathogenic organisms such as *E. coli*, *Candida albicans*, and *Staphylococcus aureus* (Silvan et al., 2013; Brenes et al., 2016). Also, Casanova-Martiì et al. (2018) demonstrated that GSPE reduced the variety of cecal microbes. Indeed, it has been established that *Lactobacilli *bacteria consume polyphenol components in GSE as nutritional substrates to metabolize phenolic compounds for delivering energy to cells and efficiently influencing bacterial metabolism (Viveros et al., 2011). Similarly, Saleh et al. (2014) reported that linalool, one of the essential oils from coriander, Ans and Grp seeds, inhibited the growth of *Escherichia coli*, *Pseudomonas aeruginosa*, *Salmonella typhimurium*, *Staphylococcus aureus*, *Clostridium botulinum,* and *Clostridium perfringens*. These results suggest that anti-microbial components of Ans and Grp oils, like coriander, may modulate the microflora in the gastrointestinal tract. Consequently, the digestibility of feed and/or growth performance in birds fed the Ans- and Grp-oil-supplemented diet might be improved.

### 4.1 Serum antioxidant activity

The effects of Ans, Grp, or their mixture on serum antioxidant indexes and lipid peroxidation as assessed by malondialdehyde (MDA) concentration and total antioxidant capacity (TAC) are shown in Fig. 2. Ans, Grp, and their mixture decreased plasma MDA levels significantly ( P≤0.01 ) compared to the control group, and the oil mixture treatment had the lowest MDA value. At the same time, no differences were found between the Ans group and the Grp group. TAC concentration was significantly increased ( P≤0.1 ) in all treatment groups compared to the control group. The Ans + Grp oil mixture had the highest TAC value, followed by the Grp oil and then the Ans oil groups. Therefore, the antioxidant enzyme activities (glutathione peroxidase, GPx) and superoxide dismutase activities (SOD) were increased significantly in all treatment groups versus the control group, and this increase was similar to the increase in TAC. Despite the influence of Grp oil on these points not being significantly different from Ans oil, the Grp oil showed an improvement in antioxidant profile compared to the Ans oil.

GSH, SOD, CAT, and GPx are essential factors of the endogenous antioxidant defense system and have a vital function in free-radical removal and maintaining the intracellular redox balance (Naaz et al., 2014). According to previous studies, GSH offers significant protection against oxidative injury by acting in the cellular defense mechanism against oxidative damage. GSH absorbs O ${}_{2}$ and prevents the oxidation of thiol groups in proteins (Ross, 1988; Jo et al., 2001). Moreover, the MDA blood content indicates lipid peroxidation within the body (Aslan et al., 2005; Enginar et al., 2006). Measuring the MDA level, which is the primary product of polyunsaturated lipid peroxidation, can determine the stage of cell damage and lipid peroxidation (Naaz et al., 2014). Increased TAC levels, decreased MAD levels in Ans- and Grp-oil-treated groups, and increased GPx and SOD enzyme activities are evidence of an improvement in the redox status balance in the quail bodies that reflects an improvement in the health and immune status of the quails. The Ans + Grp oil contains a high concentration of antioxidant compounds such as flavonoids, polyphenols, terpinenes, cumin aldehyde, monoterpene alcohols, and essential flavors (Patil et al., 2017; Saleh et al., 2019). Gagandeep (2003) proved that dietary inclusion of 2.5 % and 5 % cumin seeds enhanced the activities of superoxide dismutase and catalase in mice. Also, decreased MDA in the present results indicated that the Ans + Grp oil mixture had a high effect on enhancing TAC, GPx, and SOD components compared to the single essential oil (Ans or Grp) that plays a role in endogenous antioxidant defense against oxidative damage. The higher effect of the oil mixture than of the single oil may be due to the integration of the active substances in both Ans and Grp. Many plant-derived antioxidant compounds act as free radicals or active oxygen scavengers (Dündar, 2001).

Regarding Grp extract, in treated mice with Aflatoxin B1, GSPE increased BWG, significantly improved the spleen's oxidative damage, and alleviated the immune injury (Long et al., 2016). According to Salman (2019), adding Grp oil (at levels 1 % and 2 %) resulted in a significantly reduced malondialdehyde and peroxide value in broiler' meat compared to in the control group. By contrast, adding essential oils to quail chicks' rations in our study may be conducted to enhance the secondary lymphoid organs' immune response, along with Yazdi et al. (2014), who reported that the essential oil of aniseed could stimulate immunity.

## 5 Conclusions

Under the condition of the current experiment, Ans and Grp seed oil inclusion as a source of essential oils and natural antioxidants positively affected the growth performance, dressing percentage, and immune status. Furthermore, Ans and Grp seed oil supplementation decreased the abdominal fat yield and reduced pathogenic microbes. Thus they can replace antibiotics as a growth promoter in growing quails' diets.

## Data Availability

The corresponding author's data supporting this study's findings are available upon reasonable request.
